# Abscopal Effect of Frozen Autograft Reconstruction Combined with an Immune Checkpoint Inhibitor Analyzed Using a Metastatic Bone Tumor Model

**DOI:** 10.3390/ijms22041973

**Published:** 2021-02-17

**Authors:** Noritaka Yonezawa, Hideki Murakami, Satoru Demura, Satoshi Kato, Shinji Miwa, Katsuhito Yoshioka, Kazuya Shinmura, Noriaki Yokogawa, Takaki Shimizu, Norihiro Oku, Ryo Kitagawa, Makoto Handa, Ryohei Annen, Yuki Kurokawa, Kazumi Fushimi, Eishiro Mizukoshi, Hiroyuki Tsuchiya

**Affiliations:** 1 Department of Orthopaedic Surgery, Graduate School of Medical Sciences, Kanazawa University, 13-1 Takara-machi, Kanazawa 920-8641, Japan; nori_greenbeetle3322@yahoo.co.jp (N.Y.); skato323@gmail.com (S.K.); miwapoti@yahoo.co.jp (S.M.); kazuyashinmura@yahoo.co.jp (K.S.); chakkun1981chakkun@yahoo.co.jp (N.Y.); takaki.shimizu0928@gmail.com (T.S.); norihiron09_0820@yahoo.co.jp (N.O.); ryo415k@gmail.com (R.K.); makotohanda1020@gmail.com (M.H.); r_annen@mac.com (R.A.); kurokawa-yuki@med.kanazawa-u.ac.jp (Y.K.); tsuchi@med.kanazawa-u.ac.jp (H.T.); 2 Department of Orthopaedic Surgery, Nagoya City University Graduate School of Medical Sciences, Nagoya, Aichi 467-8601, Japan; hmuraka@med.nagoya-cu.ac.jp; 3 Department of Orthopaedic Surgery, National Hospital Organization Kanazawa Medical Center, Kanazawa, Ishikawa 920-8650, Japan; ortho0825yoshy@yahoo.co.jp; 4 Department of Gastroenterology, Kanazawa University Hospital, Kanazawa, Ishikawa 920-8641, Japan; fushimi709@gmail.com (K.F.); eishirom8848@gmail.com (E.M.)

**Keywords:** abscopal effect, frozen autograft, tumor, tumor-suppressive effect

## Abstract

We evaluated the abscopal effect of re-implantation of liquid nitrogen-treated tumor-bearing bone grafts and the synergistic effect of anti-PD-1 (programmed death-1) therapy using a bone metastasis model, created by injecting MMT-060562 cells into the bilateral tibiae of 6–8-week-old female C3H mice. After 2 weeks, the lateral tumors were treated by excision, cryotreatment using liquid nitrogen, excision with anti-PD-1 treatment, and cryotreatment with anti-PD-1 treatment. Anti-mouse PD-1 4H2 was injected on days 1, 6, 12, and 18 post-treatment. The mice were euthanized after 3 weeks; the abscopal effect was evaluated by focusing on growth inhibition of the abscopal tumor. The re-implantation of frozen autografts significantly inhibited the growth of the remaining abscopal tumors. However, a more potent abscopal effect was observed in the anti-PD-1 antibody group. The number of CD8+ T cells infiltrating the abscopal tumor and tumor-specific interferon-γ (IFN-γ)-producing spleen cells increased in the liquid nitrogen-treated group compared with those in the excision group, with no significant difference. The number was significantly higher in the anti-PD-1 antibody-treated group than in the non-treated group. Overall, re-implantation of tumor-bearing frozen autograft has an abscopal effect on abscopal tumor growth, although re-implantation of liquid nitrogen-treated bone grafts did not induce a strong T-cell response or tumor-suppressive effect.

## 1. Introduction

The antitumor immunologic effects of cryosurgery [1] were first documented by Shulman et al. [2], who demonstrated the production of antibodies against the reproductive tissues of male rabbits after freezing. Previously, we reported favorable results following reconstruction using an autograft containing a tumor subjected to cryotreatment using liquid nitrogen for primary bone tumor and bone metastasis, including spinal metastasis [3,4,5]. After en bloc resection of the tumor, the surrounding soft tissues were removed, and the specimen was immediately frozen in liquid nitrogen for 20 min. It was then gradually thawed and re-implanted with suitable internal fixation. The advantages of this method include the relative simplicity of the procedure, sufficient biomechanical strength, and the preservation of the osteoinductive potential and cartilage matrix [6,7]. In these reports, intramedullary nail, metaric plate, composite use of prosthesis, and titanilum cage were used as reconstruction and internal fixation materials. Internal fixation materials/prosthesis in the orthopedic field exhibited the inhibition effect in the formation of multinucleated cells, the production of pro-inflammatory cytokines, and the promotion in the secretion of anti-inflammatory molecules [8,9,10], and produced an anti-inflammatory microenvironment to accelerate implant ossointegration in humans [8,11]. Although tumor-bearing frozen autograft could be inferior to conventional autogenous bone grafting in terms of bone healing [12], we have considered that they may have an anti-immune effect on systemic cancers, as described below.

The abscopal effect refers to a phenomenon of tumor regression at a site distant from the primary site of radiotherapy [13]. This effect has been recorded in both case reports [13] and studies using mouse models [14,15,16] and has been reported to be possibly related to low-fractionated radiotherapy rather than single-shot radiotherapy [16,17]. In cryotreatment, the abscopal effect has also been reported in patients with prostate and breast cancer metastasis [18,19]. Previously, three cases of spinal metastasis of carcinoma and sarcoma with regression of lung or lymph node metastasis following a total en bloc spondylectomy using tumor-bearing frozen autografts for reconstruction combined with preoperative spinal embolization have been studied in our institute [4,5,20,21]. To the best of our knowledge, previous studies have used animal models in which the antitumor immune response to cryotreatment has been assessed using immune re-challenge. The effectiveness of cryosurgery alone in generating an abscopal effect is controversial. Only a few studies have examined the suppression of abscopal tumor enlargement upon cryotreatment, and it has been reported that freezing alone does not produce the abscopal effect in some cases, and specific conditions may be necessary to produce the abscopal effect. [22,23,24,25].

Recently, the effect of a combination of cryotreatment and immunotherapy has been reported [26,27,28,29,30,31,32]. In these basic and clinical research, cytotoxic T-lymphocyte-associated protein (CTLA-4) and programmed death-1 (PD-1) antagonists play a complementary role in the activation of cancer immunity, and immunotherapy followed by cryosurgery could demonstrate to induce a more cancer-specific immune response to distant lesions.

In the past, the evaluation of the antitumor effect of cryotherapy was mainly assessed by tumor rechallenge. In addition, there are no reports that have reproduced liquid nitrogen-treated bone grafting in animal models to evaluate the abscopal effect and the combination with immune checkpoint inhibitors. In this study, we evaluate the synergistic effect of PD-1 blockade in preventing the development of abscopal tumors when administered in combination with cryosurgery of a primary tumor using the murine bone metastasis model of breast cancer.

## 2. Results

### 2.1. Abscopal Tumor Growth

**Figure 1:**
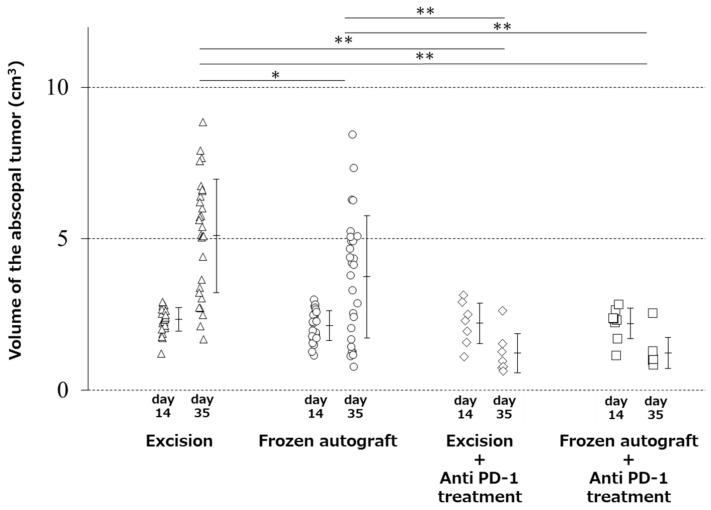
Abscopal effect of treatment on tumor volume. Image showing that the volume of the contralateral abscopal tumor at 3 weeks after treatment in the frozen autograft was significantly lower than that in the excision group (* *p* < 0.05), and the volume of the contralateral abscopal tumors treated with anti-PD-1 (programmed death-1) antibody at 3 weeks after treatment was significantly less than that of the untreated tumors (** *p* < 0.01). There was no significant difference in the tumor volume between the excision plus anti-PD-1 antibody treatment group and frozen autograft plus anti-PD-1 antibody treatment group.

The mean volume of the abscopal tumors (mean ± SD) at 2 weeks after inoculation was 2.4 ± 0.4, 2.1 ± 0.5, 2.2 ± 0.6, and 2.2 ± 0.5 cm^3^ in the excision, frozen autograft, excision plus anti-PD-1 treatment, and frozen autograft plus anti-PD-1 treatment groups, respectively. There were no significant differences in the tumor volume among the four groups (*p* = 0.48). The mean tumor volume of the contralateral abscopal tumors (mean ± SD) at 3 weeks after treatment was 5.1 ± 1.9, 3.7 ± 2.0, 1.2 ± 0.6, and 1.2 ± 0.5 cm^3^ in the excision, frozen autograft, excision plus anti-PD-1 treatment, and frozen autograft plus anti-PD-1 treatment groups, respectively (Figure 1). In this stage, there was a significant difference in the tumor volume among the four groups (*p* < 0.01). The volume of the contralateral abscopal tumor at 3 weeks after treatment in the frozen autograft group was significantly lower than that in the excision group (*p* = 0.03). However, this effect was limited compared with that in the anti-PD-1-treated groups.

The volume of the contralateral abscopal tumor at 3 weeks after treatment in the frozen autograft plus anti-PD-1 treatment group was significantly lower than that in the excision group (*p* < 0.0001) and the frozen autograft group without anti-PD-1 antibody treatment (*p* = 0.005). The volume of the contralateral abscopal tumor at 3 weeks after treatment in the excision plus anti-PD-1 treatment group was significantly lower than that in the excision group (*p* < 0.0001) and frozen autograft group (*p* = 0.0083). There was no significant difference in the volume of the contralateral abscopal tumor between the excision plus anti-PD-1 antibody and frozen autograft plus anti-PD-1 antibody groups (*p* = 1.00) at 3 weeks after treatment.

### 2.2. Abscopal Tumors from Anti-PD-1-Treated Mice Were Infiltrated by CD8^+^ T-Cells

**Figure 2:**
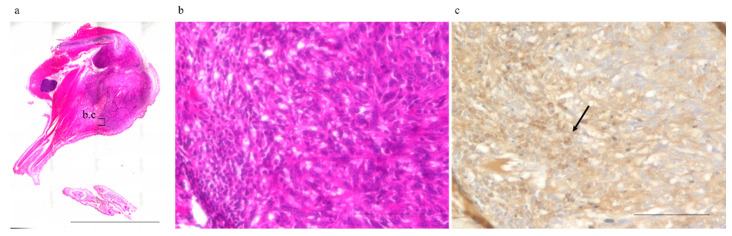
Number of infiltrating CD8^+^ T-cells surrounding the abscopal tumors. The number of infiltrating CD8^+^ T-cells surrounding the abscopal tumors was measured in the different groups. (**a**) Abscopal tumor resected 3 weeks after treatment (No. 6, frozen autograft plus anti-PD-1 treatment group). (b) and (c) show enlarged images of the area enclosed by the square. Scale bar corresponds to 10,000 µm. (**b**) Hematoxylin and eosin staining. Scale bar corresponds to 100 µm. (**c**) CD8+ T-cells (arrow) infiltrating abscopal tumors were evaluated under a high-power field by CD 8 immunohistochemical staining. Scale bar corresponds to 100 µm.

The mean number of CD8^+^ T-cells infiltrating abscopal tumors (mean ± SD), observed under a high-power field, was 13.9 ± 11.0, 18.8 ± 15.2, 40.9 ± 25.5, and 45.7 ± 21.0 cells in the excision, frozen autograft, excision plus anti-PD-1 treatment, and frozen autograft plus anti-PD-1 treatment groups, respectively (Figure 2, Table 1). There was a significant difference among the four groups (*p* < 0.01). The number of CD 8^+^ T-cells infiltrating the abscopal tumor in the frozen autograft plus anti-PD-1 antibody treatment group was significantly higher than that in the excision group (*p* = 0.0017) and frozen autograft group (*p* = 0.0119). The number of CD 8^+^ T-cells infiltrating the abscopal tumor in the excision plus anti-PD-1 antibody treatment group was also significantly higher than that in the excision group (*p* = 0.0221). There was no significant difference between the excision and frozen autograft groups (*p* = 0.8896), although the number of CD 8^+^ T-cells infiltrating the abscopal tumor tended to be higher in the frozen autograft group. Although the number of CD 8^+^ T-cells infiltrating the abscopal tumor tended to be higher in the frozen autograft plus anti-PD-1 antibody treatment group than in the excision plus anti-PD-1 antibody treatment group, there was no significant difference between the two groups (*p* = 0.9623).

### 2.3. Anti-PD-1 Treatment Prevented Splenomegaly, and Cryotreatment Alone Did Not Significantly Prevent Splenomegaly

**Figure 3:**
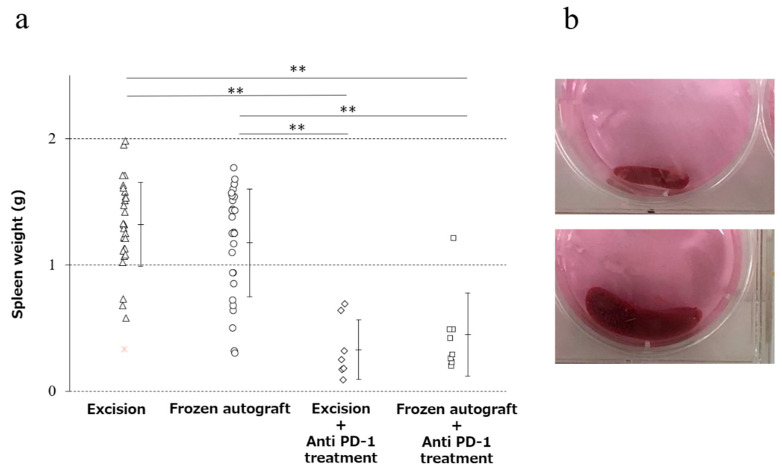
Prevention of splenomegaly in the four groups. (**a**) Diagram showing that the weight of the spleen after treatment with the anti-PD-1 antibody was significantly lower than that without anti-PD-1 antibody treatment (** *p* < 0.0001). There was no significant difference between the excision and the frozen autograft groups, although the weight of the spleen tended to be lower in the frozen autograft group. There was no significant difference between the excision plus anti-PD-1 antibody treatment and frozen autograft plus anti-PD-1 antibody treatment groups. (**b**) Normal size of the spleen (above), splenomegaly (below).

The mean weight of the spleen (mean ± SD) was 1.32 ± 0.33, 1.17 ± 0.43, 0.33 ± 0.24, and 0.45 ± 0.33 g in the excision, frozen autograft, excision plus anti-PD-1 treatment, and frozen autograft plus anti-PD-1 treatment groups, respectively (Figure 3). There was a significant difference among the four groups (*p* < 0.01). The weight of the spleen after treatment in the frozen autograft plus anti-PD-1 antibody treatment group was significantly lower than that in the excision group (*p* < 0.0001) and frozen autograft group (*p* < 0.0001). The weight of the spleen after treatment in the excision plus anti-PD-1 antibody treatment group was also significantly lower than that in the excision group (*p* < 0.0001) and frozen autograft group (*p* < 0.0001). There was no significant difference between the excision group and frozen autograft group (*p* = 0.4319), although the weight of the spleen tended to be lower in the frozen autograft group. There was no significant difference between the excision plus anti-PD-1 antibody treatment group and frozen autograft plus anti-PD-1 antibody treatment group (*p* = 0.9296).

### 2.4. Anti-PD-1 Treatment Enhanced Tumor-Associated Antigen-Specific T Cell Responses

**Figure 4:**
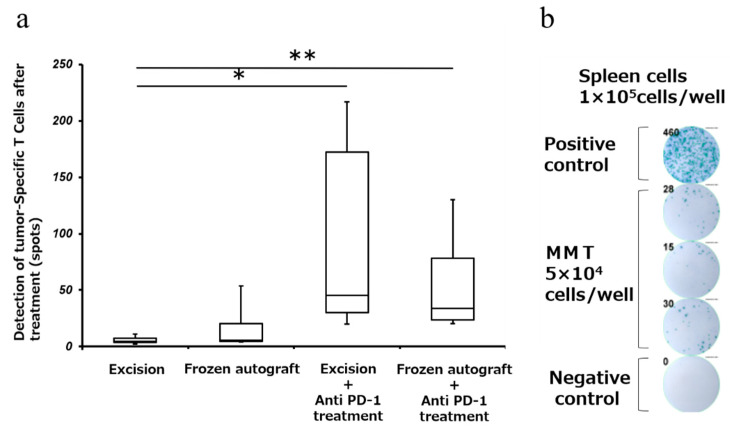
Detection of tumor-specific T cells after treatment (Interferon-γ (IFN-γ) enzyme-linked immunospot (ELISPOT) assay). (**a**) Response of tumor-specific splenocytes after treatment, using the ELISPOT assay. The number of IFN-γ-producing cells was measured using the ELISPOT assay. Diagram showing that the number of IFN-γ-producing splenocytes after treatment with the anti-PD-1 antibody was significantly higher than that in the excision group (** *p* < 0.01, * *p* < 0.05). There was no significant difference between the excision and frozen autograft groups. There was no significant difference between the excision plus anti-PD-1 antibody treatment group and frozen autograft plus anti-PD-1 antibody treatment group. (**b**) Tumor-reactive T cells were measured using the ELISPOT assay. A total of 1 × 10^5^ splenocytes were added to each well of a 96-well plate that was precoated with anti-mouse IFN-γ. MMT-060562 cells were used as stimulatory cells. After a 24 h incubation period at 37 °C, the visualized cytokine spots were enumerated using the immunospot analyzer, and the results are expressed as the number of cytokine-producing cells (No. 13, frozen autograft plus anti-PD-1 antibody treatment group).

To determine whether the tumor-specific T cells can exert effector functions, we carried out the enzyme-linked immunospot (ELISPOT) assay for interferon-γ (IFN-γ) secretion. Splenocytes were incubated with MMT-060562 cells for 24 h to induce IFN-γ secretion by tumor-reactive cells. The number of tumor-specific IFNγ-producing cells was measured using the ELISPOT assay. The mean number of spots of IFN-γ-producing splenocytes (mean ± SD) was 6.0 ± 3.9, 20.5 ± 30.0, 93.9 ± 96.3, and 59.3 ± 52.5 in the excision, frozen autograft, excision plus anti-PD-1 treatment, and frozen autograft plus anti-PD-1 treatment groups, respectively (Figure 4). There was a significant difference among the four groups (*p* < 0.05). The number of spots of IFN-γ-producing splenocytes after treatment in the frozen autograft plus anti-PD-1 antibody treatment group was significantly higher than that in the excision group (*p* = 0.0051). The number of spots of IFN-γ-producing splenocytes after treatment in the excision plus anti-PD-1 antibody treatment group was significantly higher than that in the excision group (*p* = 0.0189). There was no significant difference between the excision and frozen autograft groups (*p* = 0.69). There was no significant difference between the excision plus anti-PD-1 antibody treatment group and frozen autograft plus anti-PD-1 antibody treatment group (*p* = 0.93).

### 2.5. Splenomegaly Positively Correlated with Abscopal Tumor Development in a Metastatic Bone Tumor Model

**Figure 5:**
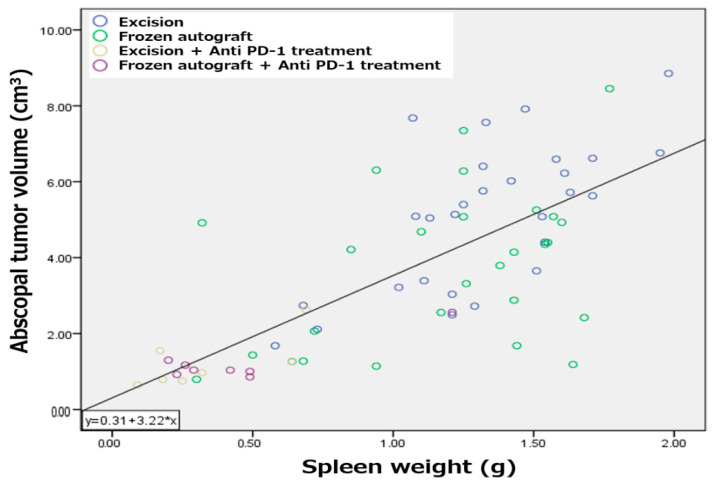
Correlation between splenomegaly and abscopal tumor development. Splenomegaly positively correlated with abscopal tumor development in a metastatic bone tumor model.

Previously, the correlation between splenomegaly and tumor development has been investigated [33]. A positive correlation between tumor development and spleen size strongly suggests that splenomegaly coincides with tumorigenesis in the MMT bone tumor model (Figure 5). The relationship between variables is expressed with the equation Y = 3.22X + 0.31, which showed a significant correlation with a Spearman correlation coefficient of 0.72.

## 3. Discussion

Recently, Waitz et al. discussed the immunological effects of cryotherapy [32]. As regression of metastases after cryotreatment was first observed in human patients, investigators have studied the immune response in animal models to demonstrate the “cryoimmunologic” effect. The results of these studies have been conflicting, and there is no consensus on the efficacy of cryotherapy alone for metastatic disease. Numerous studies have shown the immunological advantages of cryotreatments, such as cryoablation, in the rejection of secondary tumor challenge [34,35,36,37]. However, others have reported no effect [38,39] or enhancement of secondary tumor growth after cryoablation [40,41,42]. In the present study, cryotreatment alone resulted in the significant suppression of contralateral tumor growth, but it had no effect on T-cell infiltration into the abscopal tumor.

Several mediators were reported as endpoints for the abscopal effect, as reviewed by Marconi, et al. [43]. In this study, CD8-positive cells infiltrating the surround of abscopal tumors and tumor-specific IFN-producing cells in splenocytes were evaluated as mediators. We did not evaluate other mediators such as CD4 cells or dendritic cells. In this study, we employed the ELISPOT assay for tumor antigen-specific T cell responses [44]. We confirmed the abscopal effect of liquid nitrogen-treated bone grafting, but we were not able to clarify the details of the effect. Further evaluation of the mediator is needed to clarify the details of the abscopal effect of liquid nitrogen-treated bone graft.

We also compared our study with previous reports on the infiltration of CD8-positive cells into abscopal tumor. Compared to the following reports, the results in this study were comparable or slightly lower [16,45]. As shown in Figure 2, in the present study, CD8-positive cells tended to cluster at the margins of abscopal tumors and often did not infiltrate deep into the tumor. We thought that the number of CD8-positive cells might be relatively low in this study method, in which the infiltration of CD8-positive cells into the tumor was evaluated in the border region between the tumor and the surrounding normal tissue.

Furthermore, the T-cell response to tumors induced by anti-PD-1 therapy was strong, and the reimplantation of tumor-bearing frozen autografts did not activate a strong T-cell response to abscopal tumors, as achieved with anti-PD-1 therapy. This could be due to an immunosuppressive environment such as a higher concentration of regulatory T cells, which play a central role in establishing and maintaining immunological self-tolerance and homeostasis.

Tumor cells establish a microenvironment and systemic escape mechanisms (e.g., PD-L1 expression) that are conducive to tumor development and progression. Previously, a majority of cryotreatment studies used animal models, in which the antitumor immune response induced by cryotreatment was assessed by secondary tumor re-challenge. This methodology can provide an immunosuppression-free environment by removing tumor cells from the body and enabling a conducive environment for the antitumor immune response. In contrast, in an immunosuppressive environment marked by the presence of a contralateral abscopal tumor, enhancement of antitumor response induced by cryotreatment might have a limited effect, as observed in the present study. Cryosurgery with liquid nitrogen for solid tumor metastasis is mechanically similar to vaccine therapy, in which hundreds of unique antigens are released from a heterogeneous population of cancer-derived tumor cells. However, releasing tumor-derived self-antigens into circulation may not be sufficient to overcome the checkpoint escape mechanisms that some cancers have evolved to avoid immune responses.

The inhibition of PD-1 signaling via anti-mouse PD-1 treatment may sensitize tumor-specific T lymphocytes to the immunosuppressive tumor microenvironment. PD-1 is a negative costimulatory protein that is located primarily on the surface of activated CD4+ and CD8+ T-cells [46]. Signaling through PD-1 inhibits T-cells proliferation and cytokine production and attenuates cytotoxic T-cell function. PD-L1 is exclusively expressed on the surface of tumor cells and is the ligand for PD-1; their binding results in the inhibition of programmed cell death [47]. Similar to CTLA-4, PD-1 downregulates T-cell activation by turning on signaling molecules of the immune checkpoint pathway [48]. CTLA-4 and PD-1 are frequently expressed on regulatory T cells, irrespective of PD-L1 tumor expression levels. CTLA-4 and PD-1 function represent two separate regulatory pathways that mutually intersect with immune system checkpoint inhibition.

Recently, the effects of combinations of cryotreatment and immune checkpoint inhibitors have been reported [26,27,28,29,30,31,32]. Co-stimulation of the immune system with tumor antigens via cryosurgery in the presence of a “primed” immune system, pretreated with CTLA-4 and PD-1 combination therapy, would theoretically result in a synergistic effect of local tumor and distant metastasis regression or an abscopal effect [26]. In the present study, the synergistic effects of simultaneous anti-PD-1 therapy and cryosurgery were expected. Although the number of CD 8+ T-cells infiltrating the abscopal tumor in the frozen autograft plus anti-PD-1 antibody treatment group was the highest among the groups, significant synergistic effects of simultaneous anti-PD-1 therapy and cryosurgery were not observed.

Splenomegaly was positively correlated with tumor development in a murine cancer model [33,49,50]. In the present study, positive correlations between tumor development and spleen size were observed in the MMT bone tumor model. 4T1 breast cancer tumor cells reportedly induced strong splenomegaly, and an accumulation of granulocytic myeloid-derived suppressor cells in the spleen was induced in a 4T1 tumor model [51]. In the current study, splenomegaly could have occurred via the same mechanism. PD-1 knockout mice grow normally but develop splenomegaly and show an augmented proliferative B cell response and autoimmune disease [52]. Anti-PD-1 therapy might block splenomegaly and tumor development; in contrast, cryosurgery alone did not prevent splenomegaly in the MMT tumor model.

A limitation of this study is that complete re-implantation of the cryotreated tumor as a method to elicit a distant immune response was performed instead of cryoablation. It is possible that methodologies involving partial or low-dose re-implantation of frozen tumors or multiple doses of re-implantation might have resulted in a greater immune-oncological effect in distant tumors [53]. Furthermore, we were not able to investigate the immune response at multiple time points. Examination of tumor-infiltrating lymphocytes at multiple time points might provide more insight into the nature of the immune-mediated effect. There was no no-resection or no-resection plus anti-PD-1 antibody treatment group, and, therefore, it was not possible to rigorously evaluate the abscopal effect. Finally, we employed treatment regimens with check point blockade administered after cryotreatment alone, with the intent of boosting T cell activation upon antigen presentation. Minor changes in the experimental methodology could reveal a significant synergetic effect. The combination of cryotreatment and low-dose PD-1 blockade could augment the effects of checkpoint blockade in the current tumor model [29]. Further studies should explore this unique tumor microenvironment to improve tumor outcomes. If future studies reveal a synergistic relationship between anti-PD-1 therapy and re-implantation of tumor-bearing frozen autografts, more effective treatments may be established for patients with cancer metastases to the bones.

In this study, we focused on tumor-bearing frozen autograft as a treatment strategy for metastatic bone tumors. On the other hand, new treatment strategies for metastatic bone tumors, such as hyperthermia, have been also reported [54,55]. Hyperthermia of bone tumors is a promising strategy for controlling recurrence and reduces surgical side-effects, in particular, when magnetic biomaterials are used as prosthetic implants and grafts. It is hoped that further development of these studies will lead to the development of better treatments.

## 4. Materials and Methods

### 4.1. Animals

A total of 74 six–eight-week-old female C3H mice were purchased from Sankyo Labo Inc. (Toyama, Japan). This study was approved by the Committee of Animal Care and Experimentation at Kanazawa University (Kanazawa, Japan, AP-143308). The animals were maintained in cages (3–6 mice per cage) under natural illumination with free access to water and food. The mice were anesthetized using an intraperitoneal injection of pentobarbital sodium (0.05 mg/kg body weight). All surgeries were performed with the mice under sodium pentobarbital anesthesia, and all efforts were made to minimize suffering to the mice. Following each procedure, the mice were monitored in their cages until they recovered from anesthesia. The health of the mice was monitored daily during this study. The mice were euthanized by intraperitoneal overdose injection of pentobarbital sodium.

### 4.2. Tumors

A murine breast cancer cell line, MMT-060562, derived from a spontaneous mammary tumor in a C57BL x A/F1 hybrid female mouse, was provided by the European Collection of Authenticated Cell Cultures (ECACC) Cell Lines, Australia. These cells were maintained in complete medium consisting of D-MEM (High Glucose) with L-glutamine and phenol red (Wako Pure Chemical Industries, Osaka, Japan) supplemented with 10% heat-inactivated fetal bovine serum, 100 μg/mL streptomycin, and 100 units/mL penicillin, and cultured at 37 °C in 5% CO_2_. The breast cancer cell line used in this study, MMT-060562, was selected because it is a naturally occurring breast cancer cell line that can easily generate bone metastasis models in non-immunodeficient mice and is useful for immunological validation [56,57]. The metastatic potential of MMT was low, and it was necessary to create abscopal tumors separately. We also thought it would be useful to evaluate the pure abscopal effect. A limitation is that we could not reproduce the naturally occurring bone metastasis model.

### 4.3. Procedure

**Figure 6:**
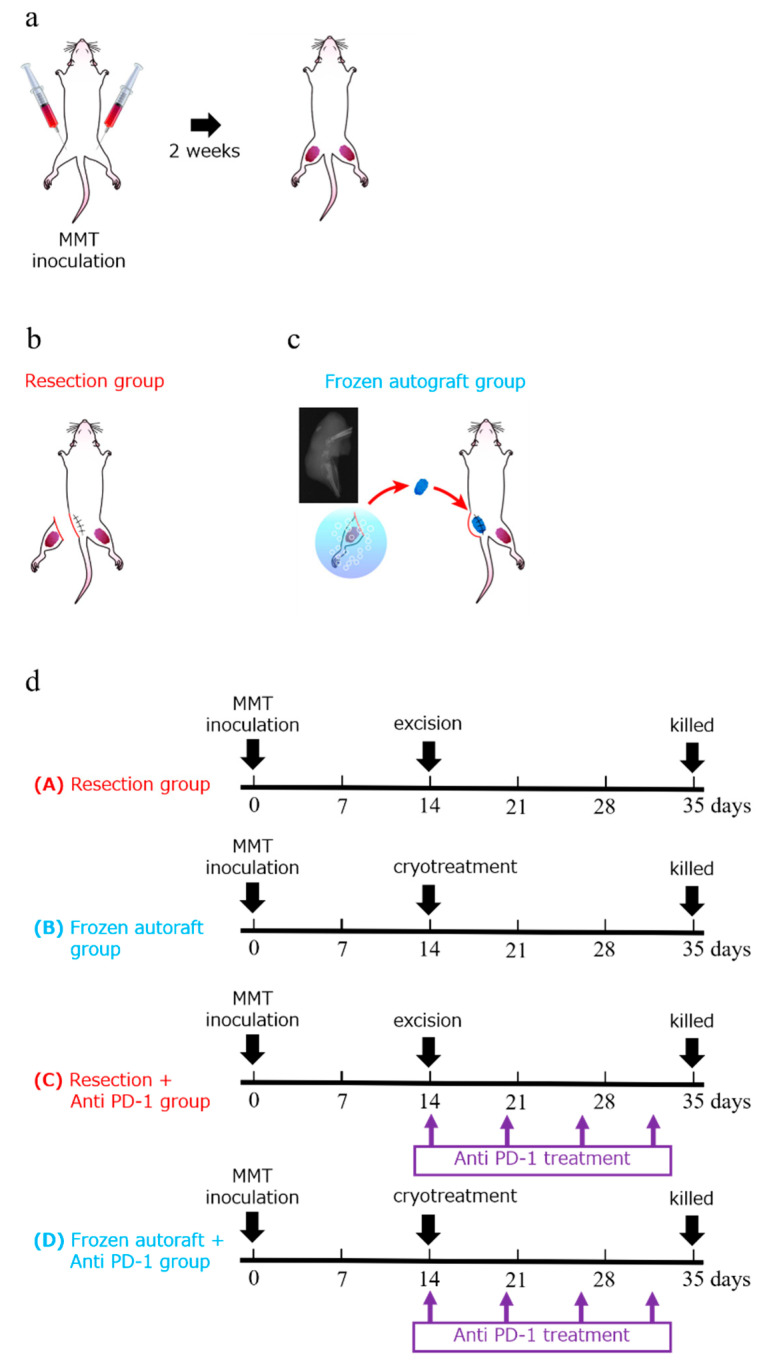
Experimental schema and diagram showing the experimental protocol and treatment schedule. (**a**) Experimental schema of an approximately 5 mm midline skin incision made to expose the tibial tuberosity. To establish bilateral bone metastasis of the tumor, 3 × 10^6^ MMT cells were suspended in 50 μL of Matrigel and injected into the bilateral tibia of the mice. (**b**–**d**) Two weeks after the injection, the tumors were treated using one of the following methods: (**a**) excision, (**b**) cryotreatment using liquid nitrogen, (**c**) excision with intraperitoneal injection of anti-PD-1 antibody after treatment, and (**d**) cryotreatment using liquid nitrogen with intraperitoneal injection of anti-PD-1 antibody after treatment. The mice were intraperitoneally injected with anti-mouse PD-1 4H2 on days 1 (20 mg/kg), 6 (10 mg/kg), 12 (10 mg/kg), and 18 (10 mg/kg). Five weeks after the injection, the mice were euthanized and evaluated.

To establish bilateral bone metastasis of the tumor, a 5 mm midline skin incision was made to expose the bilateral tibial tuberosity, and 3 × 10^6^ MMT cells suspended in 50 μL of Matrigel (BD Biosciences, San Jose, CA, USA) were injected into the bilateral tibia of the mice (Figure 6a). Two weeks after the injection, the lateral tumors were treated as follows: (a) excision, (b) cryotreatment using liquid nitrogen, (c) excision with intraperitoneal injection of anti-PD-1 antibody after treatment, and (d) cryotreatment using liquid nitrogen with an intraperitoneal injection of anti-PD-1 antibody after treatment. Excisions were performed with grossly negative surgical margins. After controlling the hemorrhage, the wound was closed using interrupted nylon sutures in the excision groups. In the cryotreated groups, after en bloc excision of the tumor, the surrounding soft tissues were partially removed, and the tumor tissue was collected on gauze and soaked in liquid nitrogen (−196 °C) for 20 min for en bloc freezing. The tissue was pre-thawed at room temperature (20 °C) for 15 min and thawed in distilled water (20 °C) for 15 min. The freezing and thawing procedures were performed safely according to the method reported by Yamamoto et al. [6]. It was then subcutaneously transplanted into the same leg of the same animal, and the wound was sutured (Figure 6b,c).

### 4.4. Immune Checkpoint Inhibitor for Adjuvant Treatment

Mice treated with surgical excision or liquid nitrogen were intraperitoneally injected with anti-mouse PD-1 4H2 (Ono Pharmaceutical Co., Ltd. Osaka, Japan) on days 1 (20 mg/kg), 6 (10 mg/kg), 12 (10 mg/kg), and 18 (10 mg/kg) after treatment (Figure 6d).

### 4.5. Evaluation of Abscopal Effect

**Figure 7:**
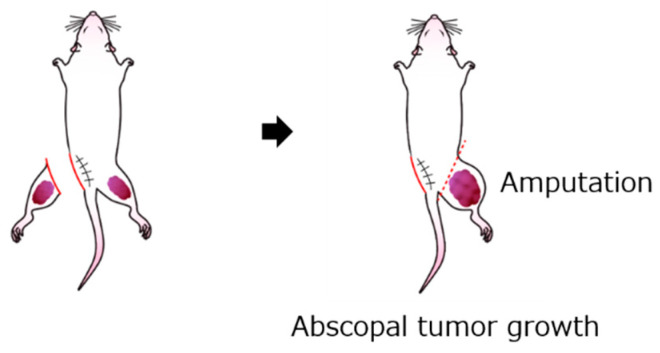
Schema for measuring abscopal effect and immunological response. The contralateral abscopal tumor volume was evaluated at 14 and 35 days after tumor cell injection.

The abscopal effect was evaluated by measuring the untreated contralateral abscopal tumor volume in the excision (*n* = 28), frozen autograft (*n* = 27), excision plus anti-PD-1 treatment (*n* = 7), and frozen autograft plus anti-PD-1 treatment (*n* = 8) groups. The contralateral abscopal tumor volumes were measured at 14 and 35 days after tumor cell injection. The abscopal tumor diameter was measured on days 14 and 35 after tumor cell injection. The abscopal tumor volume was calculated using the following formula: Tumor volume (cm^3^) = (smallest diameter)^2^ × largest diameter × 1/2 [58]. The mice were euthanized on day 35 after inoculation. The untreated contralateral limb containing the abscopal tumor was resected, and the tumor tissue of the resected limb was evaluated (Figure 7).

### 4.6. Infiltrating CD8^+^ T-Cells Surrounding the Abscopal Tumors

CD8^+^ T-cells were subjected to immunohistochemical staining. Each tissue sample from the contralateral limb containing the abscopal tumor was embedded in paraffin and sectioned to 4 μm thickness. A rabbit polyclonal antibody against CD8 (Bioss; bs-0648R; 1:200) was used as the primary antibody. Anti-mouse or rabbit IgG conjugated with peroxidase-labeled polymers (EnVision, Dako, Carpinteria, CA, USA) was used as the secondary antibody. The number of infiltrating CD8^+^ T-cells surrounding the abscopal tumors was measured in the excision (*n* = 13), frozen autograft (*n* = 11), excision plus anti-PD-1 treatment (*n* = 5), and frozen autograft plus anti-PD-1 treatment groups (*n* = 7). In the four groups, photographs of the border regions of the abscopal tumor and normal tissue were acquired. For each analysis, fifteen independent high-power fields were randomly selected for each mouse. The number of CD8^+^ T-cells surrounding the abscopal tumors was counted using a BZ-9000 microscope (KEYENCE, Osaka, Japan). Slides were coded, slide identification was blinded, and positive cells for CD8 were counted by N.Y. All mice were evaluated uniformly with the same resolution and clarity. The mean value of CD8-positive cells in one high-power field of each mouse was summed and divided by the total number of mice to obtain the mean value and standard deviation.

### 4.7. Evaluation of Spleen Weight

The mice were euthanized on day 35 after inoculation. The spleen was gently excised under aseptic conditions, and the spleen weight was measured in the excision (*n* = 28), frozen autograft (*n* = 27), excision plus anti-PD-1 treatment (*n* = 7), and frozen autograft plus anti-PD-1 treatment groups (*n* = 8).

### 4.8. Tumor-Specific IFN-γELISPOT Assay

The number of tumor-specific IFN-γ-producing splenocytes was measured using the ELISPOT assay. Briefly, a 96-well plate was coated with the anti-mouse IFN-γ antibody (Cellular Technology Ltd., Cleveland, OH, USA). Activated splenocytes (1 × 10^5^ cells/well) were cultured for 24 h at 37 °C in a 5% CO_2_ incubator alone or in the presence of MMT-060562 tumor cells (5 × 10^4^ cells/well). Thereafter, the wells were washed and incubated with a biotinylated anti-IFN -γ antibody. Reactions were visualized and spots were counted using anti-biotin-AP after 24 h of incubation at 37 °C, using an immunospot analyzer (Cellular Technology Ltd., Cleveland, OH, USA), and the results are expressed as the number of cytokine-producing cells. The ELISPOT assay was performed for excision (*n* = 8), frozen autograft (*n* = 7), excision plus anti-PD-1 treatment (*n* = 6), and frozen autograft plus anti-PD-1 treatment groups (*n* = 8).

### 4.9. Selected Experimental Endpoints and the Large Tumor Volumes in This Study: Statement

The experimental endpoint of 5 weeks from tumor administration to the final evaluation was considered a realistic setting, considering that multiple doses are required to obtain reliable effects with respect to the levels of anti-PD-1 antibodies. In this experiment, a constant tumor size was required to compare the changes in tumor size over time in each group. We considered that insufficient tumor engraftment and growth could occur in mice due to variations in the tumor injection technique and other factors. We were concerned that this could reduce the accuracy and reproducibility of the experiment, especially when the volume of the injected tumor was small. Therefore, we ultimately used a large tumor volume as an endpoint because the site of tumor implantation was neither a major organ nor the abdomen. Tumors were grafted into the limbs and, therefore, were less likely to result in fatal organ damage. This experiment was reviewed and approved by the Animal Care and Experimentation Committee of Kanazawa University. The mice were periodically observed after surgery. In fact, only a few mice were removed from the experiment as they were weak or for other reasons. The mice were euthanized and treated humanely.

### 4.10. Statistical Analysis

Mean differences between the groups were statistically evaluated using non-repeated measures ANOVA, followed by Tukey–Kramer test. Statistical significance was set at *p* < 0.05. Statistical analysis was performed using JMP version 11 (SAS, Carey, NC, USA) and SPSS statistical software version 23 (SPSS, Chicago, IL, USA).

## 5. Conclusions

Re-implantation of a tumor-bearing frozen autograft exerted an abscopal effect. The T-cell response to tumors induced by anti-PD-1 therapy was strong, and re-implantation of a tumor-bearing frozen autograft did not induce a strong T-cell response to abscopal tumors as achieved with anti-PD-1 therapy. This study presents promising results of liquid nitrogen-treated tumor-bearing bone grafting in bone metastases of malignant tumors for inhibiting the progression of systemic cancer.

## Figures and Tables

**Table 1:** Number of CD 8^+^ T-cells infiltrating the abscopal tumor observed under a high-power field in the four groups.

Group	Number of CD8^+^ T-Cells Infiltrating the Abscopal Tumor
Excision	13.9 ± 11.0
Frozen autograft	18.8 ± 15.2
Excision plus anti-PD-1 treatment	40.9 ± 25.5 *
Frozen autograft plus anti-PD-1 treatment	45.7 ± 21.0 **^,#^

* *p* < 0.05 for the excision group, ** *p* < 0.01 for the excision group, ^#^ *p* < 0.05 for the frozen autograft group.

## Data Availability

All relevant data are within the paper.
